# Basics of Radiation Biology When Treating Hyperproliferative Benign Diseases

**DOI:** 10.3389/fimmu.2017.00519

**Published:** 2017-05-03

**Authors:** Franz Rödel, Claudia Fournier, Julia Wiedemann, Felicitas Merz, Udo S. Gaipl, Benjamin Frey, Ludwig Keilholz, M. Heinrich Seegenschmiedt, Claus Rödel, Stephanie Hehlgans

**Affiliations:** ^1^Department of Radiotherapy and Oncology, University Hospital of Frankfurt, Goethe-Universität, Frankfurt am Main, Germany; ^2^Department of Biophysics, GSI Helmholtz Centre for Heavy Ion Research, Darmstadt, Germany; ^3^Department of Radiation Oncology, Universitätsklinikum Erlangen, Friedrich-Alexander-Universität Erlangen-Nürnberg, Erlangen, Germany; ^4^Department of Radiotherapy, Clinical Center Bayreuth, Bayreuth, Germany; ^5^Strahlenzentrum Hamburg MVZ, Hamburg, Germany

**Keywords:** low-dose radiation therapy, hyperproliferative diseases, fibroblasts/myofibroblast, cytokines, antiproliferative effect, anti-inflammatory effect

## Abstract

For decades, low- and moderate-dose radiation therapy (RT) has been shown to exert a beneficial therapeutic effect in a multitude of non-malignant conditions including painful degenerative muscoloskeletal and hyperproliferative disorders. Dupuytren and Ledderhose diseases are benign fibroproliferative diseases of the hand/foot with fibrotic nodules and fascial cords, which determine debilitating contractures and deformities of fingers/toes, while keloids are exuberant scar formations following burn damage, surgery, and trauma. Although RT has become an established and effective option in the management of these diseases, experimental studies to illustrate cellular composites and factors involved remain to be elucidated. More recent findings, however, indicate the involvement of radiation-sensitive targets like mitotic fibroblasts/myofibroblasts as well as inflammatory cells. Radiation-related molecular mechanisms affecting these target cells include the production of free radicals to hamper proliferative activity and interference with growth factors and cytokines. Moreover, an impairment of activated immune cells involved in both myofibroblast proliferative and inflammatory processes may further contribute to the clinical effects. We here aim at briefly describing mechanisms contributing to a modulation of proliferative and inflammatory processes and to summarize current concepts of treating hyperproliferative diseases by low and moderate doses of ionizing radiation.

## Introduction

The capacity of ionizing radiation to inhibit proliferation of malignant cancer cells are well explored (1–3) and widely used in clinical practice. By contrast, application of radiation therapy (RT) for non-malignant conditions is not a fully accepted practice in medicine. In line with that, the use of RT in the management of hyperproliferative non-cancerous disorders is controversially discussed and inadequately recognized by doctors from disciplines others than RT. However, long-term experiences impressively indicated a clinical benefit for patients (4, 5). Accordingly, treatment with irradiation concepts not exceeding a single dose of 5 Gy and total doses of 30 Gy [low- or intermediate-dose RT (LD-RT)] is an established and effective modality in the management of a variety of non-cancerous inflammatory, degenerative, and hyperproliferative/fibroproliferative disorders (4–6). The latter include, among others, heterotopic ossifications, symptomatic vertebral hemangiomas, Gorham–Stout syndrome, prophylaxis of keloid relapse after surgical excision (7), and, most prominent, palmar and plantar fibromatosis also known as Dupuytren disease (DD) and Ledderhose disease (LD) (8). The most effective treatment schedule, the radiobiological basis, and molecular/cellular mechanisms contributing to the modulation by ionizing radiation of these benign hyperproliferative disorders are far from being fully explored. Consequently, this review aims at summarizing current clinical concepts and antiproliferative as well as immune modulating properties of low- and moderate-dose irradiation focusing on DD, LD, and keloids. This may display a prerequisite for future systematic investigations to enhance clinical irradiation protocols.

## Use of RT to Treat Benign Disorders

Non-malignant indications for LD-RT comprise about 10–30% of all patient cases treated in most academic, public, and private RT facilities in Germany (4, 9). In total, more than 50,000 patients per year are treated by LD-RT with the largest group suffering from painful degenerative musculoskeletal diseases, followed by symptomatic functional and hyperproliferative disorders with the latter to increase in numbers by 28.8% from 1999 to 2004 (8).

In 1831, Baron Guillaume Dupuytren described for the first time a fibrotic contracture of the palmar fascia of the hand, while fibrotic contractures of the plantar fascia of the foot were initially described by the German physician Georg Ledderhose in 1897 (4). DD is a prevalent disease with incidences varying between populations with up to 29% in the Western countries (10). Men are affected more often and earlier in life as women with a gender ratio from 3:1 to 6:1 (11) and with an onset of symptoms usually in the third to fourth decade of life (12). Concerning the etiology and pathogenesis of DD, several studies report on a strong genetic background (13, 14) apart from environmental risk factors including alcohol, smoking, hand trauma, and manual work (15–17).

In spite of a documented occurrence of 1.75 cases per 100,000 hospital admissions, the precise incidence of LD remains not exactly specified (18). It is known that men are affected twice as often as women, and in 25% of patients, both feet are involved. In 9–25% of patients, concomitant DD has been described (19, 20), while a coincidence with knuckle pads or Peyronies’s disease has been observed in 4% of cases (21).

Another clinically relevant example of benign hyperproliferative diseases are keloids, which are considered as dermal disorders in predisposed individuals caused by injuries to the deep dermis, including burn damage, surgery, and trauma. The classic description of a keloid is “an exuberant scar formation that extends beyond the borders of the original wound.” Keloids are relatively common diseases occurring in 5–15% of wounds (22) and tend to affect both sexes equally. The frequency of keloid occurrence in persons with highly pigmented skin is 15 times elevated compared to those with less pigmented skins (23). Surgical resection is the standard in treating keloid patients, but excision alone results in unacceptably high recurrence rates of 45–100% (24).

According to a recent guideline from the German Society of Radiation Therapy and Oncology (DEGRO), single doses of 0.5–1.0 Gy (total doses of 3.0–6.0 Gy) and two or three fractions per week are recommended in patients with painful degenerative and inflammatory diseases (6, 8). By contrast, different schedules are advised when treating hyperproliferative diseases like DD, LD, and keloids (5, 25). So far, total doses exceeding 20 Gy applied in single fractions of 3 Gy have been shown to comprise the most clinically relevant schedules. However, at present, only a few controlled studies have reported on alternative fractionation concepts. Against this background, a randomized study comparing no treatment versus either 21 or 30 Gy applied in 3-Gy single fractions over 2 weeks (7 Gy × 3 Gy) or by repeated 5 Gy × 3 Gy at intervals of 12 weeks has been conducted in patients with DD. After a median follow-up of 8 years, both regimes were significantly superior regarding disease progression and avoidance of preceding surgery compared to the control group (9). In a huge retrospective cohort, Betz et al. further analyzed a total of 135 DD patients (208 hands) treated with a total dose of 30 Gy, in two intervals of 5 daily fractions of 3.0 Gy, separated by 6–8 weeks. At a median follow-up of 13 years, early-stage disease was more likely to respond to treatment in terms of prevention of progression (26), and 66% of the patients showed a long-term relief of symptoms, while RT was not associated with increased complications following salvage surgery in case of progression and late skin toxicity (atrophy, dry desquamation).

In contrast to DD, only a few clinical investigations have been published concerning RT of LD. After a median follow-up of 22 months, Heyd et al. reported a complete remission of the nodes in 33.3% of cases and a decrease or numerical reduction in 54.5% of the cases following weekly fractions of 3.0 Gy (15 Gy), repeated after 6 weeks. About 70% of the patients indicated a reduction of pain and an improvement of their gait pattern (18).

As mentioned before, keloid scars tend to display high recurrence rates of 45–100% following surgical debulking or resection (24). By contrast, adjuvant RT has been shown to result in the avoidance of renewed excessive scar formation and good cosmetic outcome with a 60–90% success rate (22, 27, 28). There is conclusive evidence that single doses of 2.0–5.0 Gy and total doses of 16–20 Gy/series with five fractions per week are effective for the prevention of local relapses after surgical excision of keloids (5). RT can be applied with low-energy X-rays (150–200 kV), low-energy electrons (4–10 MeV), or brachytherapy (29). To obtain the optimal antiproliferative effect, radiation should be initiated immediately after the surgical excision, preferably within the first 24 h.

In conclusion, the clinical/empirical experience of different dose requirements and treatment schedules to treat degenerative and hyperproliferative benign diseases may indicate distinctive cellular components and mechanisms to be affected in response to ionizing radiation. In case of hyperproliferative disorders, both antiproliferative and anti-inflammatory effects may account for elevated dose requirements that will be reviewed below.

## Basic Mechanisms of Radiation Exposure and Cancer Risk Assessment after RT of Benign Diseases

In the last decades, there has been increasing interest in the physical and molecular cellular response following exposure to ionizing radiation. Initial events cover damage to DNA by direct hits of photons or electrons or generation of radicals, e.g., reactive oxygen species (ROS), that indirectly cause DNA double-strand beaks (DSBs), the most severe kind of damage (30, 31). Induction of these lesions promptly results in the activation of DSB damage repair processes, most importantly non-homologous end joining or homologous recombination, and subsequently triggers execution of a multitude of cellular signaling pathways referred to as the DNA damage response (DDR) (2, 32). These responses cover posttranslational modifications and/or altered gene expression of proteins to initiate cell cycle alterations (e.g., radiation-induced arrest) or execute cell death by mitotic catastrophy, apoptosis, autophagy, or induction of senescence (2, 3, 32). Importantly, the classical paradigm in radiobiology on (nuclear) targeted effects, indicating that DNA DSBs are solely responsible for the biological consequences of radiation exposure, is now challenged by reports on non-(DNA) targeted effects. These effects cover, among others, bystander or abscopal effects and adaptive responses and are considered to be involved in the regulation of intercellular communication and modulation of the activity of a multitude of immune components by low- or intermediate-dose ionizing radiation [reviewed in Ref. (33)]. Accordingly, although not proven experimentally at present, one may assume that RT of hyperproliferative disorders may include both targeted (cell proliferation/death) and non-targeted effects of ionizing radiation (modulation of immune components).

Due to reports from the sixties of the last century on increased mortality from leukemia and anemia (34), LD-RT is still considered unfashionable in some countries. However, risk assessment of carcinogenesis after low-dose radiation treatment of benign diseases is challenging due to a relatively small number of patients treated worldwide, latency of carcinogenesis, which requires a long-term follow-up, and different treatment regimes and techniques that are not directly comparable with the present advanced methodology (35, 36). In general, the risk to develop radiation-induced cancer can be estimated by calculation of the equivalent dose of a specific tissue or organ using the effective dose (E) concept as proposed by the International Commission of Radiological Protection (37). These estimations, however, are controversially discussed and problematic in cases where organs receive heterogenous exposure, and calculation of the effective dose might overestimate the true probability in some cases and underestimate it in others (38). An alternative and more accurate approach for the estimation of the risk to develop malignancies is a direct assessment from epidemiological data of patients who have undergone radiotherapy for benign diseases (35, 38). However, these data are still scarcely available, and follow-up times are often too short. In summary, estimation of cancer risk after radiation treatment for benign diseases is challenging, but for current clinical protocols regarded to be small especially for older patients (36). By contrast, the balance of risk and benefit has to be considered carefully for younger patients, and children should not be subjected to LD-RT at all.

## Cellular and Molecular Basis of Hyperproliferative Diseases

Dupuytren disease and LD are among the best-described diseases with proliferation of fibrous tissue to form two structurally distinct elements, nodules and cords, which have features in common with benign fibromatosis (39, 40). Aberrant cellular proliferation is involved in the formation of these elements, which are induced by a genuine unknown reason, injury, or a variety of trigger mechanisms (41). Histologically, nodules present a highly vascularized tissue with a high percentage of fibroblasts and myofibroblasts, while cords are more avascular, acellular, and collagen-rich tissues. As mentioned before, the prominent cellular components in the nodules are fibroblasts/myofibroblasts. The latter comprise differentiated cells that share characteristics of fibroblasts and, by the expression of α-smooth muscle actin, contractile properties similar to those of smooth muscle cells (15, 42, 43). These myofibroblasts originate from several sources including quiescent tissue fibroblasts, circulating cluster of differentiation (CD)34+ fibrocytes, and a phenotypic conversion of various cell types including epithelial and endothelial cells.

Several studies further indicated infiltration of multiple immune cells in Dupuytren’s contractures. These cover different lineages of lymphocytes including CD3, CD4, CD8, CD45RA+ naïve and CD45RO+ activated cells, CD68- and S100-positive macrophages (44), and Langerhans cells. Further, compared to peripheral blood detection, transcription factor FOXP3-positive regulatory T-cells were more abundant in fibrotic tissue. Notably, immunoscope analysis indicated a restricted T-cell receptor αβ repertoire, indicating an (auto)antigen-driven expansion of intralesional T-cell clones with Th1-/Th17-weighted immune responses (44). Finally, in favor of a causal involvement of inflammatory processes in DD, elevated levels of the pro-inflammatory cytokines interleukin (IL)-6 and an abundant expression of transforming growth factor-β1 (TGF-β1) have been reported (44).

In contrast, keloids present reddish tumor-like lesions extending beyond a surgical scar (28), which do not respect the borders of the original wound area. Functionally, keloids arise from either insufficient degradation and remodeling of extracellular matrix (ECM) components due to an imbalance in expression of matrix metalloproteinases or excessive ECM deposition by an increased activity of fibroblasts and myofibroblasts (45). Furthermore, keloid stem cells have been described, which share characteristics with skin progenitor cells and are transformed from dermal progenitor cells in a pathological niche of keloid tissues. These keloid stem cells are self-renewal and, by asynchronous divisions, continually generate new keloid cells, thus leading to overgrowth of keloid tissue and posttherapy recurrences (46).

Recently, to assess characteristics of cellular composition, tissue specimens from 28 keloid patients were subjected to immunohistochemical analyses (47). An increased number of CD20- and CD3-positive lymphocytes, CD68-positive macrophages, and CD1α+ Langerhans cells were recorded, indicating characteristics in keloid tissue similar to autoimmune diseases (47). This notion was further strengthen by the detection of elevated levels of TGF-β1; vascular endothelial growth factor (VEGF); platelet-derived growth factor-α in line with inflammatory cytokines IL-6, IL-8, and IL-18; and chemokine-like factor 1 (48, 49).

## Proliferating Mitotic Fibroblasts/Myofibroblasts are Radiosensitive Cells

Concerning radiation responsiveness, the course of DD and LD comprises three consecutive phases. These include a radiosensitive initial hyperproliferative period characterized by increased numbers of fibroblasts/myofibroblasts in line with an excessive deposition of ECM components, especially collagen, fibronectin, elastin, and proteoglycans (50, 51). The initial period is followed by an involutional phase with decreased radiation sensitivity in line with the formation of fiber bundles causing contractures. Finally, this phase is followed by a non-RT responsive residual phase with a predominant establishment of collagen filaments in the connective tissue (4, 42). Thus, the clinical implementation and clinical efficacy of RT to treat hyperproliferative DD and LD are strictly stage dependent, with a clinical efficacy most pronounced in the early nodular stage. With regard to target cells, the proliferative phase is characterized by the presence of radiation-responsive fibroblasts and/or myofibroblasts preceding the formation of nodular contractures (52, 53). These myofibroblasts differentiate from fibroblasts triggered by activation with fibrogenic cytokines secreted by macrophages or other cellular compounds (15, 51). This differentiation/activation process results in proliferation and excessive production of ECM components as mentioned before (54). The cellular source(s) of these myofibroblasts are still not entirely clear; however, they may be multiple (55). In addition to resident mesenchymal cells, myofibroblasts are derived from epithelial or endothelial cells in a process termed epithelial–mesenchymal transition or endothelial–mesenchymal transition (56–58). Moreover, a unique circulating fibroblast-like cell derived from bone marrow stem cells (59, 60) further accounts for myofibroblast development. These blood-born mesenchymal progenitors have a fibroblast/myofibroblast-like phenotype as they express CD34, CD45, and type I collagen and are commonly called fibrocytes.

Notably, in the field of radiation biology, an alternative definition of fibrocytes exists that differs from the immunological one given above that may cause some confusion. In their reports, Bayreuther and Rodemann indicated fibrocytes to constitute terminally differentiated postmitotic fibroblasts (PMF) with downregulation of transcription factor c-fos and a specific capacity for the synthesis of collagen types I, III, and V and proteoglycans (39, 61, 62). Taking this definition into account, single-dose irradiation in the range of 1–8 Gy has been shown to induce terminal differentiation of these cells into senescent fibrocytes at a high percentage level. By contrast, irradiation of long-term cultures with repeated doses of 10 times 0.6 Gy or 10 times 1.0 Gy revealed a marked reduction of their proliferative capacity (63, 64). This has even been demonstrated for densely ionizing irradiation (65). In line with that, the life span of non-proliferating PMF is limited and shortened by more than 40% following irradiation in comparison to physiological conditions (66). Moreover, these populations require a permanent renewal from a mitotically active progenitor fibroblast pool (67). Consequently, interference with the differentiation processes in line with eradicating mitotic precursor fibroblasts may display a substantial fundament for the clinical effects of antiproliferative low-dose irradiation.

From a mechanistic point of view, RT results in reduction of fibroblast proliferation, cell cycle arrest, and induction of cellular senescence as has been shown in irradiated long-term cultures of healthy human fibroblasts. Following an immediate cell cycle arrest, a period of a few weeks with premature differentiation and senescence was observed (68). Inhibition of cell proliferation and induction of cellular senescence were mediated by interruption of the cell cycle with an extended GO/G1 phase, in line with upregulation of cell cycle regulators TP53 and CDKN1A (p21) and senescence-associated genes p16 and p27 at protein levels (68, 69). Notably, concerning radiation-induced cell death, primary lung fibroblasts were able to prevent radiation-induced apoptosis by activation of protein kinase C (PKC), while PKC inhibition or attenuation results in downregulation of prosurvival and antiapoptotic signaling proteins and apoptosis induction (70).

Another study investigated the effect of irradiation on primary keloid fibroblasts (KFb) (71). X-ray exposure inhibited KFb proliferation and induced cell senescence in a dose- and time-dependent manner. On a molecular basis, mRNA and protein expression of senescence-associated genes p16, p21, and p27 increased after 4 Gy irradiation in a time-dependent manner. Responsible for this is considered a dynamic feedback-loop, triggered by activation of p21, followed by mitochondrial dysfunction and increased levels of ROS, resulting in elevated DNA damage and ongoing DDR (72). However, the fate of the fibroblast after irradiation-induced cell cycle arrest is not only determined by persistent DNA damage and p21 levels but also essentially depends on cellular Cdk2/p21 ratio (73).

## Impairment of Proliferative Activity of Fibroblasts/Myofibroblasts by Free Radicals

It is a well-established fact that levels of ROS including superoxide (O^2−^), hydrogen peroxide (H_2_O_2_), and hydroxyl radical (⋅OH) dramatically increase following exposure to ionizing radiation, resulting in damage to macromolecules and DNA in line with disturbance of a multitude of signal transduction pathways (74–77). These pathways, in a direct way, stimulate production of inflammatory and fibrogenic mediators that include chemotactic cytokines, mitogens, and mediators to modulate differentiation of the fibroblast/myofibroblast/fibrocyte axis (78, 79). Accordingly, the microenvironment in contracture tissue is characterized by the presence of a multilevel network of inflammatory/fibrogenic cytokines, ROS, and antioxidants that in sum may interfere with the clinical effectiveness of LD-RT. A close connection between ROS production and local ischemia was further confirmed in an early study showing elevated quantities of hypoxanthine and xanthine oxidase activity to catalyze elevated levels of O^2−^ and H_2_O_2_ in palmar fascia of patients with DD (80). Besides this, addition of free oxygen radicals to cultures of fibroblasts derived from DD palmar fascia dose dependently increases collagen type III expression at low concentrations or inhibits proliferation at higher doses (81). This possibly may indicate that ionizing radiation induces a level of ROS production that exceed a threshold to inhibit proliferation of fibroblasts and/or myofibroblasts.

## Cytokines and Growth Factors Comprise Targets of Radiation in Hyperproliferative Diseases

Analogous to inflammatory diseases and fibrotic disorders, levels of cytokines and growth factors secreted by a multitude of cell types including platelets and macrophages have extensively been analyzed in DD, LD, and keloid specimens (82–84). These molecules cover fibroblast growth factor, PDGF, epidermal growth factor, connective tissue growth factor, TGF-β1, IL-1, IL-6, VEGF, and tumor necrosis factor-α (TNF-α) (41, 83, 85–87). TGF-β1 is well documented to constitute a key player (84, 88), which is undoubtedly among the cytokines most implicated in both the process of fibrosis induction and radiation response. TGF-β1, which is produced by a wide range of inflammatory, mesenchymal, and epithelial cells, is critical in many facets of the fibrogenic process, such as ROS generation and conversion of fibroblasts into myofibroblasts (43, 86, 89). The factor transduces its signal by a heteromeric complex formation of related type I and type II transmembrane receptors, resulting in phosphorylation and activation of receptor-regulated mother against decapentaplegic homolog 2 (Smad2) and Smad3 molecules (R-Smads). These R-Smads in turn associate with Smad4 (Co-Smad) to form a heteromeric Smad transcription factor complex that regulates expression of a large array of target genes (90). All of these components were reported to have increased expression patterns in DD, resulting in accelerated TGF-β signaling (88, 91). Importantly, Wong and Mudera further reported on a negative feedback inhibition of TGF-β1 in Dupuytren’s fibroblasts. In their study, the group reported on lower doses (1–10 ng/ml) to increase myofibroblast activation in an experimental collagen model, whereas higher concentrations (20–30 ng/ml) impaired contraction in DD fibroblasts (92). Accordingly, it is convincible to assume that increased TGF-β1 transcription and secretion triggered by ionizing radiation in endothelial cells and fibroblasts/fibrocytes (18, 63, 64) may result in inhibition of fibroblast/myofibroblast proliferation and ECM deposition in irradiated tissue.

More recently, TNF-α was identified as an additional key regulator involved in the fibrotic process and differentiation of fibroblasts into myofibroblasts in the palm of patients affected by DD, *via* activation of Wnt signaling pathway (13, 87). Moreover, TNF-α directly regulates TGF-β1 expression, as shown in lung fibroblasts (93). Finally, targeting TNF-α by the use of neutralizing antibodies diminished the contractile activity of myofibroblasts derived from DD patients, reduced the expression of α-SMA, and mediated disassembly of the contractile apparatus, thus qualifying the cytokine as a therapeutic target in DD.

## Impact of Macrophage Activity and Endothelial Cells on Proliferation of Myofibroblasts

While factors affecting the beginning and development of DD and LD as well as keloids have been extensively studied (15, 25, 82, 94), the mechanistic basis for the regulation of proliferative elements remains not entirely resolved. These processes, however, may include several prominent elements: a fibrogenic/angiogenic element associated with proliferation and an immune cell component. Indeed, histological studies identified the presence of clusters of macrophages and T-lymphocytes in early DD and keloids and a correlation between the numbers of macrophages and the quantity of myofibroblasts (87, 95, 96).

**Figure 1:**
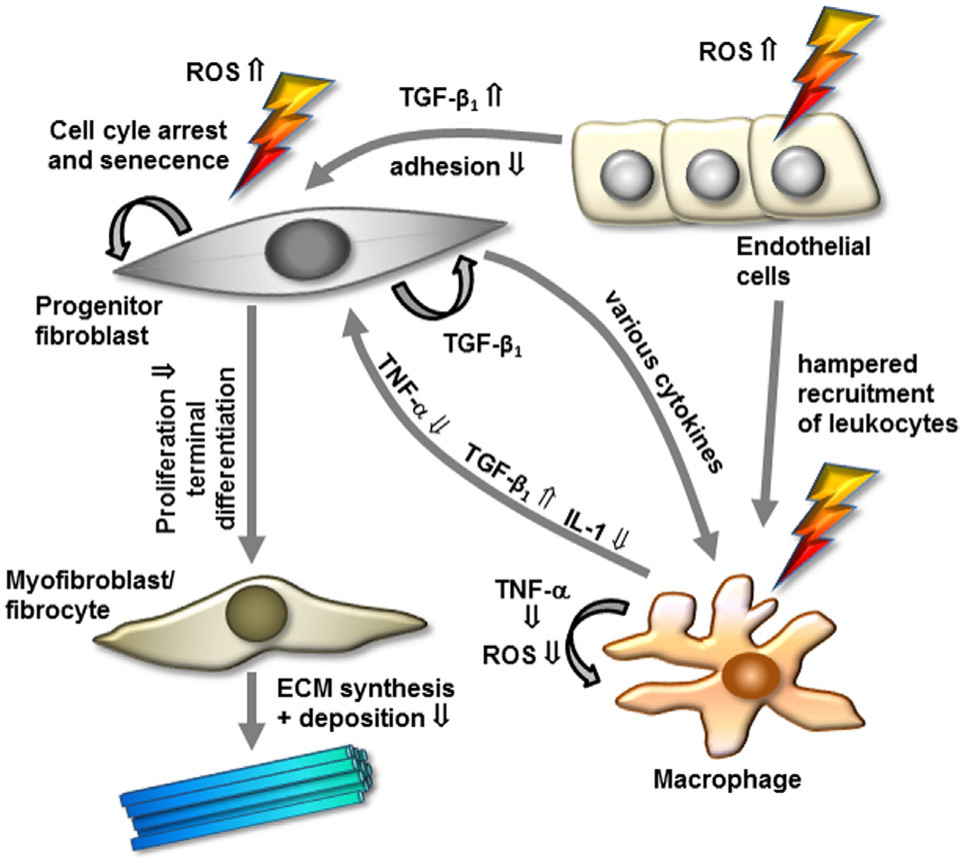
**Model of modulation of cellular components and factors by low-dose radiotherapy for the treatment of hyperproliferative/fibrotic benign diseases**. Progenitor mitotic fibroblasts are activated by transforming growth factor-β1 (TGF-β1) and additional factors to differentiate into myofibroblasts/fibrocytes, resulting in increased extracellular matrix (ECM) synthesis and deposition. In contrast, irradiation might interfere with these processes by increasing free radicals, inactivating radiosensitive mitotic fibroblasts/myofibroblasts, and promoting terminal differentiation into senescent fibrocytes. Further, low-dose irradiation modulates inflammatory components in modulating cytokine expression, macrophage, and endothelial cell activity. Abbreviations and details are given in the text.

Notably, with regard to cytokine production, a hampered pro-inflammatory TNF-α and IL-1 secretion from human RAW 264.7 or murine macrophages stimulated by lipopolysaccharides has been reported following LD-RT (97–99). Mechanistically, the hampered cytokine production was correlated to a diminished nuclear translocation of the immune relevant transcription factor nuclear factor kappaB (NF-κB) subunit RelA (p65) in line with a lowered induction of NF-κB upstream p38 mitogen-activated protein kinase and downstream protein kinase B (Akt) (99, 100). In addition, inflammatory macrophages revealed a reduction in their capacity to perform an oxidative burst and a diminished activity of the enzyme inducible nitric oxide synthase upon low-dose irradiation, resulting in lower levels of ROS and nitric oxide (NO) induction (101, 102). Considering the pivotal function of macrophages in inflammatory and fibrogenic cascades, a lowered production of cytokines, ROS, and NO may essentially contribute to a hampered myofibroblast proliferation and to the clinical benefit of low- and intermediate-dose irradiation in hyperproliferative disorders (Figure 1).

It further has been shown that a clinically therapeutic effect of steroids if given in an early phase of DD, results from a reduction in leukocyte adhesion/diapedesis (103) as well as increased apoptosis of macrophages and fibroblasts (104). In a mechanistic manner, endothelial cells are critically implicated in the regulation of (pro-)inflammatory cascades, which are mediated by a locally restricted adhesion of immune components from the peripheral blood and secretion of an array of cytokines/growth factors including TGF-β1 and IL-6 (105–107). In that context, our group and others have shown a diminished leukocyte adhesion to 40–50% of the level of non-irradiated cells most pronounced at a 4- and 24-h period following LD-RT. This effect is mainly mediated and functionally attributed to the expression of TGF-β1 from endothelial cells (106, 108, 109). Accordingly, it is reasonable to speculate that a hampered recruitment of monocytes/macrophages from peripheral blood may promote antiproliferative/inflammatory properties of low- and intermediate-dose ionizing radiation and thus contributes to beneficial effects of LD-RT in DD, LD, and keloids.

## Conclusion and Future Perspectives

The pathogenesis of hyperproliferative/fibrogenic disorders is complex, considered to evolve from system biology diseases based on a multitude of (patho)physiological networks (110), and still remains elusive despite extensive investigation. Accordingly, one may assume that the empirically proven beneficial efficacy of (low dose) RT is mediated by the modulation of a variety of pathways and cellular targets involved (Figure 1). Among these targets, the fibroblast/myofibroblast system originating from several sources comprises a characteristic connector, linking DD, LD, and keloid diseases. Radiation-related molecular mechanisms affecting these cellular components include a direct influence on cell cycle regulation, production of oxygen radicals to diminish their proliferative capacity, and interference with growth factor and cytokine expression (15). Moreover, reduced numbers of activated immune cells implicated in concomitant inflammatory processes, and proliferation of fibroblasts/myofibroblasts (111, 112) may further contribute to the therapeutic effects of radiation. Consequently, the use of low- or moderate-dose RT for early-stage DD and LD and postsurgical keloids not only covers a robust radiobiological rationale but also has been proven as low-cost and effective treatment with clinically acceptable acute and long-term toxicity (8). Even though remarkable progress has been achieved during the last years in the knowledge of radiobiological mechanisms most prominent after a low-dose exposure (33, 113), a therapeutic efficacy in hyperproliferative disorders may originate from an overlap of antiproliferative and immune-modulatory effects as documented by different dose requirements in daily clinical applications.

As stated before, the number of patients annually treated with low- and intermediate-dose irradiation at least in Germany continuously increases in line with a growing acceptance from other medical disciplines. Moreover, based on preclinical radiobiological considerations (113), recent trials confirmed a clinical isoeffect of single dose of 0.5 and 1 Gy irradiation (total dose 3 or 6 Gy) in terms of pain relief and long-term response at least in degenerative skeletal disorders (114, 115). Consequently, for radiation protection purposes and decreasing putative radiation risk, standard use of 0.5 Gy/3 Gy schedules is now recommended for the treatment of these diseases (6). Although comparable optimization studies are still lacking in hyperproliferative disorders, one may draw the conclusion by analogy that a dose reduction may further increase acceptance of RT in the clinical management of DD, LD, and keloids and increase numbers of patients treated for these indications worldwide. Moreover, in terms of a decrease in single and total doses, combined modality treatment with, e.g., anti-inflammatory drugs should be addressed in future clinical investigations to boost treatment routines including RT.

Very recently, a modular assay for detailed immunophenotyping of peripheral whole blood samples of patients following low-dose radon spa therapy (RAD-ON01 study) (116, 117) and low-dose X-irradiation (IMMO-LDRT01: http://ClinicalTrials.gov identifier: NCT02653079) have been developed. These multicolor flow cytometry approaches may be well adapted for a detailed monitoring of immunological properties in patients with DD, LD, and keloids. Accordingly, to the author’s point of view, future research activities should concentrate on basic, translational, and clinical efforts (dose optimization studies, patient’s immunophenotyping, and combined modality treatment) and on the development of suitable preclinical models for hyperproliferative disorders to further characterize additional factors and mechanisms contributing to the clinical effects of LD-RT.

## Author Contributions

FR, CF, JW, FM, UG, BF, CR, and SH: drafted the manuscript and wrote it together with the coauthors and performed final evaluation. LK and MS: drafted the manuscript (clinical part) and wrote it together with the coauthors and performed final evaluation.

## Conflict of Interest Statement

The authors declare that the research was conducted in the absence of any commercial or financial relationships that could be construed as a potential conflict of interest.
